# The Role of Selenium and Manganese in the Formation, Diagnosis and Treatment of Cervical, Endometrial and Ovarian Cancer

**DOI:** 10.3390/ijms241310887

**Published:** 2023-06-29

**Authors:** Anna Golara, Mateusz Kozłowski, Paweł Guzik, Sebastian Kwiatkowski, Aneta Cymbaluk-Płoska

**Affiliations:** 1 Department of Reconstructive Surgery and Gynecological Oncology, Pomeranian Medical University in Szczecin, Al. Powstańców Wielkopolskich 72, 70-111 Szczecin, Poland; mtkoozo@gmail.com; 2 Clinical Department of Gynecology and Obstetrics, City Hospital, 35-241 Rzeszów, Poland; pawelguzik@gmail.com; 3 Department of Obstetrics and Gynecology, Pomeranian Medical University in Szczecin, Al. Powstańców Wielkopolskich 72, 70-111 Szczecin, Poland; kwiatkowskiseba@gmail.com

**Keywords:** selenium, manganese, gynaecological, nanoparticles, endometrial cancer, ovarian cancer, cervical cancer

## Abstract

Selenium (Se) and manganese (Mn) are essential micronutrients that are important elements of cell metabolism. They are involved in the composition of enzyme systems and regulate enzyme activity. Disturbances in the homeostasis of these micronutrients affect the development of many diseases and carcinogenesis, which can be linked to increased levels of oxidative stress and impaired antioxidant properties of many enzymes. Selenium has a very important function in maintaining immune-endocrine, metabolic and cellular homeostasis. Manganese, on the other hand, is important in development, digestion, reproduction, antioxidant defense, energy production, immune response and regulation of neuronal activity. We review the role of selenium and manganese and their effects on tumor growth, metastasis potential and remodeling of the microenvironment. We also describe their role as potential biomarkers in the diagnosis and the potential for the use of Se- and Mn-containing compounds in composition for the treatment of cancer of the reproductive organs.

## 1. Introduction

Selenium (Se) and manganese (Mn) are micronutrients that are involved in cellular metabolism, act in enzyme systems and regulate enzyme activity. In biological systems, these elements are mostly associated with proteins [1,2]. Deficiency of any of them leads to undesirable pathological conditions, which can be prevented or reversed by appropriate supplementation. In sufficiently nourished individuals, supplementation should be carefully controlled because of its toxic effects when present in quantities above physiological standards [3,4]. Micronutrient levels depend on environmental factors such as air pollution, diet and lifestyle habits such as smoking and caffeine consumption. Mn cations and Se anions have unpaired electrons that enable them to participate in redox reactions. Most of the biological effects, mainly toxic, of these elements can be explained by their ability to catalyse the initiation of free radical reactions or the breakdown of peroxides and other unstable molecules [5,6]. Low selenium levels result in an increased risk of mortality, weakened immune system function and impaired cognitive function. Selenium deficiency is also associated with hypothyroidism, subclinical hypothyroidism, thyroid enlargement [7], thyroid cancer [8], Hashimoto’s disease (HT) [7] and Graves’ disease (GD) [9,10]. In contrast, higher selenium levels or selenium supplementation has antiviral effects, affects female and male reproduction and reduces the risk of autoimmune thyroid disease. Numerous studies have shown the beneficial effects of higher selenium levels on the risk of prostate, breast, lung, esophageal and gastric cancer [11,12]. However, additional selenium intake may only benefit people with low selenium levels, people with adequate and high selenium levels may be adversely affected and should not take selenium supplements as this may, for example, increase the risk of type 2 diabetes [13,14,15].

This review summarises the biological roles of selenium and manganese in homeostasis, proliferation and apoptosis. We review their roles and interdependencies in oncogenesis and formation of cancers of the ovary, cervix and endometrium.

## 2. Selenium

Selenium is a micronutrient required for the proper functioning of the body. It is a non-metal that can occur in inorganic form in the form of selenates (VI) and selenates (IV), or in organic form in the form of the amino acids selenomethionine and selenocysteine [16]. These amino acids were formed by replacing the sulphur atom present in cysteine and methionine with a selenium atom. In selenocysteine, there is a selenium group (-SeH) instead of a thiol group (-SH), while in selenomethionine, there is a selenium atom at the 4th carbon instead of a sulphur atom. They build the important enzymes for the human body: glutathione peroxidases, thioredoxin reductases, iodothyronine deiodinases and 12 individual selenoproteins such as selenoprotein P [17]. Their functions in the body are described in (Table 1). Selenoproteins have different subcellular locations, have diverse functions and are regulated in distinct pathways. They have an important function in maintaining immune-endocrine, metabolic and cellular homeostasis [18].

Glutathione peroxidases are mainly responsible for the reduction in hydrogen peroxide (H_2_O_2_) or organic peroxides using reduced glutathione as an electron donor [19]. Thioredoxin reductases reduce thioredoxins and protein disulfides and are involved in DNA synthesis. They also influence the process of apoptosis. Glutathione peroxidases and thioredoxin reductases mainly have antioxidant properties—they protect the cell from the damaging effects of reactive oxygen species [20,21]. Iodothyronine deiodinases are involved in the thyroid hormone conversion reaction. The best-known and most important selenoprotein is selenoprotein P [22]. It is produced in hepatocytes and its activity has been reported in various tissues of the body including plasma. It consists of two domains: a larger N-terminal domain that maintains the redox potential in the cell and a smaller C-terminal domain that mediates selenium transport [23]. Selenoprotein P also protects lipoproteins from oxidation [24].

### 2.1. Selenium Homeostasis

The main sources of selenium are meat, fish and cereal products such as pasta and oatmeal. In contrast, the food containing the most selenium is Brazil nuts [25]. The best-absorbed form of selenium is its organic form in the form of the amino acid selenocysteine, which is absorbed in the small intestine [26]. The metabolism of selenium depends on the form in which it enters the body. It can be absorbed in organic and inorganic forms. The inorganic forms are selenate (VI) and sodium selenite (selenate IV), while the organic forms are selenomethionine and methylated selenocysteine [17].

**Figure 1:**
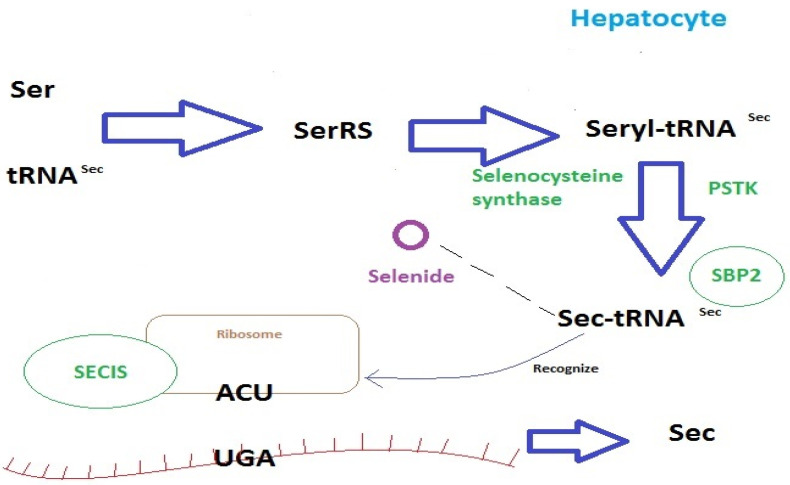
Sec biosynthetic pathway. tRNA^Sec^—selenocysteine-specific tRNA; SerRS—seryl-tRNA synthetase; PSTK—O-phosphoseryl-tRNA^Sec^ kinase; SBP2—Sec 2 insertion sequence binding protein; SECIS- Sec insertion sequence.

Selenium in inorganic form is reduced by glutathione and NADPH, while selenium derived from assimilated selenomethionine is incorporated into cysteine and selenocysteine is formed. Selenomethionine can also be incorporated into proteins in place of methionine [27]. The main metabolite of these reactions is selenohydrogen, and it is the border point between two pathways of selenium metabolism in the body. The first pathway is the metabolism of inorganic compounds with selenohydrogen as the end product. In the second pathway, selenohydrogen is the starting point for the biosynthesis of selenoproteins [28]. This pathway involves the cotranslational biosynthesis of selenocysteine and its incorporation into selenoproteins by tRNA. The reaction begins with the attachment of serine by seryl-tRNA synthase, and then selenocysteine synthase catalyses the replacement of the hydroxyl group of serine with a selenol moiety, which is derived from selenium phosphate. These reactions produce a tRNA that binds selenocysteine. The insertion of selenocysteine is determined by the UGA codon, which is responsible for terminating translation. Sec-tRNA modification thus allows selenocysteine insertion into the resulting protein [29]. The specific Sec biosynthetic pathway is shown in Figure 1.

### 2.2. Glutathione Peroxidase

Human glutathione peroxidases exist in the form of eight isoenzymes (GPX1-8), five of them (GPX1-4 and 6) are selenocysteine (Sec)-containing proteins (SecGPX) and three (GPX5, 7 and 8) have cysteine (CysGPX). These isoenzymes are encoded by different genes and differ in molecular structure, subcellular localisation, substrate specificity and biological function. Peroxidases catalyse the reduction in H_2_O_2_ or organic hydroperoxides to water or alcohols using glutathione (GSH) as a reductant [30]. They also take part in regulatory processes and have synthetic functions. Glutathione peroxidases do not have a defined Michaelis constant or substrate saturation [31], therefore achieve infinite reaction rates at infinite concentrations of reaction substrates [32]. GPXs are involved in a variety of diseases, including neurodegenerative disorders, diabetes, cardiovascular disease and cancer [33].

Glutathione peroxidase (GPx1) was discovered as the first selenoprotein. It uses glutathione to catalyse hydrogen peroxide, lipid peroxidation and peroxynitrite, reducing intracellular oxidative stress [34]. The GPX1 gene is located on chromosome 3p21.31 with 1178 base pairs and contains two exons. Selenium, which is found in the GPX1 protein as a selenoprotein, is formed by the insertion of the 21st amino acid selenocysteine into the polypeptide chain during the translational coding process at the stop codon UGA. The UGA stop codon sequence completes translation in proteins that do not encode selenocysteine, but the 3′ UTR of the selenoprotein mRNA contains a stem-and-loop cis-element, otherwise known as the Sec insertion sequence (SECIS) or Sec insertion sequence, which is necessary for the recognition of UGA as a Sec codon rather than as a stop codon [35]. GPX1 has a catalytic tetrad consisting of Sec or Cys, glutamine (Gln), tryptophan (Trp) and asparagine (Asn). Sec in the GPX1 protein is flanked by four arginines and one lysine from the neighbouring subunit [30]. The GPX1 gene is regulated at the transcriptional, post-transcriptional and translational levels.

The human GPX1 gene has several genetic polymorphisms, the most common being a cytosine (C) to thymine (T) substitution in exon 2 at codon 198 (Pro198Leu, dbSNP ID: rs1050450), which changes proline (Pro, CCC) to leucine and GPX1 drops by 5%. There are three genotypes in the population according to the GPX1 Pro198Leu polymorphism: Pro/Pro homozygotes, Pro/Leu heterozygotes and Leu/Leu homozygotes [36]. The substitution of Pro for Leu is thought to lead to a conformational change in the GPX1 protein which may affect tumour susceptibility [37]. The GPX1 Pro198Leu polymorphism is being intensively studied in various types of cancer [38], urinary bladder [39], breast cancer [40], lung [41], prostate [42], colon [43] and leukaemia [44]. Meta-analyses do not provide a clear answer as to whether the GPX1 Pro198Leo polymorphism can promote cancer susceptibility [45,46].

GPX1 influences the development of various human malignancies, patient survival and prognosis [47]. It may participate in various signalling pathways that regulate tumour development, including cell proliferation, apoptosis, invasion, immune response and chemo-resistance. However, the expression levels and prognostic values of GPX1 in different tumour types are still controversial according to different cohorts and statistical analyses. GPX1 overexpression can eliminate reactive oxygen species, inhibit apoptosis and induce drug resistance, promoting cancer cell survival [48,49,50]. 

The most potent and best-described GPX1 inhibitor is mercaptosuccinic acid (MSA), which competes with glutathione for the Sec GPX1 active site [51]. Pentathiepins, on the other hand, have a much stronger inhibitory activity on the GPX1 enzyme than MSA. It induces loss of mitochondrial membrane potential and oxidative stress in cancer cells, leading to DNA strand breaks and apoptosis [52]. Combining GPX1 inhibitor treatment with photodynamic therapy can generate synergistic anti-tumour effects by enhancing oxidative stress, accumulating ROS and inducing apoptosis in cancer cells [53].

GPx2 peroxidase is mainly found in the gastrointestinal tract including the oesophageal epithelium and, in humans, also in the liver. It acts as a barrier against the absorption of hydroperoxides from food [54]. The function of this enzyme is to protect against lipid peroxides resulting from lipid peroxidation. The highest concentration of GPx2 protein occurs at the base of the crypts, which gradually decreases to the top of the crypts in the colon or to the top of the crypts in the large intestine or to the villi in the small intestine, respectively [55].

GPx3 peroxidase is an extracellular enzyme that is actively released into the plasma. It is responsible for the reduction in superoxides in plasma and other body fluids. Glutathione peroxidase GPx4 reduces H_2_O_2_ and small hydroperoxides in complex lipids such as phospholipid, cholesterol and cholesterol hydroperoxides [56] and is found in many tissues.

### 2.3. Antioxidant Properties

Reactive oxygen species (ROS) include superoxide (O_2_¯) and hydrogen peroxide (H_2_O_2_), which are produced in aerobic metabolism. They play an important role in cellular signal transduction and homeostasis. Unfortunately, if ROS levels increase and exceed the capacity of cellular antioxidant defence systems, they can cause significant damage to cellular structure and function, known as oxidative stress [57].

Glutathione peroxidases catalyse the reduction in H_2_O_2_ and lipid hydroperoxides by converting glutathione (GSH) to oxidised glutathione (GSSG), thereby protecting the body from oxidative damage [19]. The reaction of the glutathione peroxidases GPx1, GPx3 and GPx4 is a three-substrate reaction following a ping-pong mechanism. The kinetics of GPx cannot be described as Michaelis kinetics with constant Vmax and KM. The catalytic cycle can be divided into a peroxidation part and a reduction part. In the peroxidation part, the oxidation of selenol in GPx to selenic acid by hydroperoxide (ROOH) occurs. The first GSH forms selenium disulfide with selenic acid, while oxygen is removed as H_2_O. The second GSH reduces selenium disulfide in a thiol-disulphide reaction. Thus, GSSG is released and the enzyme regenerates to the selenium form which can be used in the next cycle [58].

### 2.4. Selenium and Oncogenesis

Selenium has antioxidant properties, but can also act as a prooxidant to be toxic to cancer cells [59,60]. Cancer cells have an abnormal metabolism that involves increased production of endogenous reactive oxygen species (ROS). Increased levels of ROS induce increased oxidative stress. Cancer cells maximise their antioxidant capacity to become more susceptible to additional levels of ROS.

Induction of ROS production by Se through redox modulation is a promising therapeutic strategy to selectively kill cancer cells [61]. Se compounds are selectively toxic to cancer cells, especially chemotherapeutic drug-resistant cells, exhibiting biological activity through active redox metabolites. Two well-characterised and widely used Se compounds are methylselenic acid (MSA; CH_3_SeO_2_H) and sodium selenite (Na_2_SeO_3_).

In Paper [62], Enqvist et al. showed that selenite (SeO₃²¯), an inorganic selenium compound, induces a near loss of HLA-E expression on the surface of cancer cells of various origins, which increases the susceptibility of CD94/NKG2A-positive NK cells to kill cancer cells. CD94/NKG2A is an inhibitory receptor that controls the activity of a large proportion of human NK cells. In contrast, Hagemann–Jensen et al. [63] showed that MSA alters the expression of NKG2D ligands on tumor cells, thereby influencing better recognition and elimination by NKG2D-expressing immune effector cells.

### 2.5. Selenium Supplementation

According to European recommendations, selenoprotein P (SEPP1) is an indicator of adequate selenium supply to all tissues and reflects the saturation of the functional selenium pool in the body, ensuring that selenium requirements are met. The adequate intake (AI) for adults is 70 µg/day. For infants aged 1–7 months, the AI is at 15 µg/day. AI values range from 15 µg/day for children aged one to three years to 70 µg/day for adolescents aged 15–17 years. The AI set for adult women applies to pregnancy. However, for breastfeeding women, an additional selenium intake of 15 µg/day was estimated to cover the amount of selenium secreted in breast milk and the AI was set at 85 µg/day [64].

Adequate levels of bioavailable selenium are important for the proper functioning of the central nervous system, male reproductive system, endocrine system, muscle function, cardiovascular system and immunity.

### 2.6. The Importance of Selenium Supplementation for Cancer Treatment

Reduced selenium levels in serum and tumour tissue are observed in patients with cervical cancer [65,66]. Se plays an important role in the human immune system, the proliferation of B lymphocytes and the increased function of T lymphocytes, but is also an essential component of antioxidants such as the glutathione peroxidase enzyme and thioredoxin reductase. Antioxidants control intracellular peroxide levels in the mitochondria and cytoplasm and help protect the cell from oxidative damage from reactive oxygen species released to kill engulfed bacteria. However, the mechanism of action of Se as an anticancer agent is not yet sufficiently understood. Exposure to selenium may be a protective factor for cervical cancer. HPV infection is one of the common risk factors for cervical cancer, and high serum selenium levels may reduce the risk through an antiviral role [67]. Therefore, selenium supplementation may reduce the risk of cervical cancer [68]. Taking Se supplements for 6 months by women with CIN1 leads to its regression and has beneficial effects on markers of insulin metabolism, TAG, VLDL and HDL-cholesterol levels, as well as biomarkers of oxidative stress [69]. In addition, Se supplementation during radiotherapy to the pelvic region effectively improves blood Se status in patients with cervical cancer and reduces the incidence and severity of RT-induced diarrhea [70,71].

There is a significant association between lower serum selenium levels with the incidence of endometrial cancer [72]. However, the discussion on selenium supplementation in the prevention of endometrial cancer is still ongoing [73].

Low serum Se levels are observed in patients with malignant ovarian tumors [74]. The observed reduction in Mn and Se levels in erythrocytes may be related to an adaptive defence mechanism of tumour cells either directly or through Mn-SOD and GPx1 to prevent OS-induced or caspase-induced apoptosis in epithelial ovarian cancer (EOC) from increasing. Mn levels may be important for differentiating tumour staging, and loss of Mn may contribute to tumour growth and/or spread. Additionally, the observed increase in Cu/Se ratio may be responsible for the reduced antioxidant capacity of the blood and increased inflammatory response due to low Se levels in all histological subtypes of EOC. Thus, a high Cu/Se ratio in erythrocytes may be a favourable marker for EOC [75]. Some studies suggest that a higher intake of supplemental selenium may be inversely related to ovarian cancer risk [76], especially in women with BRCA1 mutations. BRCA1 mutation contributes to an increase in 8-oxodG in cellular DNA, which may be a factor responsible for the development of cancer in women with the mutation. However, oxidative DNA damage and the risk of breast cancer development in BRCA1 mutation carriers may be reduced in patients who have undergone adnexectomy with selenium supplementation [77]. Selenium supplementation in the form of selenium-enriched yeast in ovarian cancer patients who are being treated with chemotherapy increases the concentration of this micronutrient in the body, which activates selenium-dependent enzymes and reduces the adverse effects of chemotherapy [78].

Because of the observed correlation between selenium levels and the incidence and course of cervical, endometrial and ovarian cancers, we suggest manganese supplementation according to European guidelines and continuing research in this direction.

## 3. Manganese

Manganese is a micronutrient essential for normal bone development, macronutrient metabolism and defence against ROS. It acts as a cofactor for various enzymes including arginase (rate-limiting enzyme for urea synthesis), glutamine synthetase (GS) (critical for ammonia metabolism in the brain), acetyl-CoA carboxylase (critical catalyst for endogenous fatty acid synthesis), phosphoenolpyruvate decarboxylase and pyruvate carboxylase (gluconeogenesis), Mn superoxide dismutase (mitochondrial antioxidant) and glycosyltransferase (bone health) [79]. Mn is transported in the body by transferrin and by macroglobulins and albumin.

Metalloproteins have important functions in development, digestion, reproduction, antioxidant defence, energy production, immune response and regulation of neuronal activity. Mn deficiency is rare but can cause birth defects, fertility disorders, bone malformations and increased vulnerability [80]. In contrast, Mn poisoning can occur with overexposure to this metal and happens more frequently. Mechanisms of toxic effects of Mn on the body include oxidative stress, mitochondrial dysfunction, abnormal protein folding, endoplasmic reticulum (ER) stress, dysregulation of autophagy, apoptosis and disruption of homeostasis of other metals.

Excess Mn usually accumulates in the liver, pancreas, bones, kidneys and brain, which can cause cirrhosis, polycythaemia, hypermagnesemia, extrapyramidal symptoms resembling idiopathic Parkinson’s disease (PD). Most patients present with neurological symptoms and liver dysfunction with microglandular cirrhosis and elevated transaminases and unconjugated hyperbilirubinaemia [81]. Manganese in toxic doses can also interfere with cardiovascular function, causing an abnormal electrocardiogram, increased heartbeat, shorter P-R interval and reduced diastolic blood pressure [82].

### 3.1. Manganese Homeostasis

Dietary sources of Mn include rice, nuts, whole grains (wheat germ, oats, bran) and legumes, also green leafy vegetables, tea, chocolate and seafood (clams) are also rich in Mn. Drinking water contains Mn at levels ranging from 1 mg/L to 2 mg/L, depending on location and contamination [83].

Manganese is absorbed mainly from the gastrointestinal tract but also through inhalation and skin penetration, and then transported to target tissues via the bloodstream [84]. Finley et al. [85] conclude that manganese metabolism differs between men and women, and suggest that different levels of iron may be the cause. Mn is absorbed in the intestines by passive diffusion or by active transport. Mn transport in intestinal cells is biphasic with saturation similar to other divalent cations [86]. The divalent metal transporter 1 (DMT1) is responsible for the active transport of Mn and other divalent cations, so the presence of other metals competing for the same transporter regulates Mn absorption. Patients with low Fe levels are at higher risk of Mn poisoning, as intestinal absorption of Mn can increase under conditions of low Fe concentration [87]. Lönnerdal et al. [88] suggest that adding Ca to human milk significantly reduces Ca absorption in adult men and women. In contrast, Davidsson et al. [89] say that adding phytate, phosphate and ascorbic acid to infant formula and iron and magnesium to wheat bread does not significantly affect Mn absorption. Another factor that affects Mn absorption is age. Infants and children absorb a higher amount of Mn from the diet because of the high need for Mn for body development [90].

In the human body, the liver, pancreas, bones, kidneys and brain are the organs containing the highest levels of Mn. It is absorbed through the gastrointestinal tract or lungs and then transported to various tissues through the bloodstream. In contrast, excess Mn is conjugated to bile by the liver and excreted in the feces [91]. Therefore, people with liver problems have a higher risk of Mn poisoning.

### 3.2. Manganese and Oncogenesis

Reduced manganese levels are observed in cervical and ovarian cancers, which can be used in the diagnosis and treatment of these cancers. Strong expression of manganese superoxide dismutase (MnSOD) is also present in cervical cancer. 

There are also many studies describing manganese-containing compounds that can be used in cancer diagnosis. Polyethyleneimine-coated manganese oxide nanoparticles may be used in the future for positron emission tomography (PET)/magnetic resonance (MR) imaging of tumors [92]. Au/MnO_2_/ERGO/CF nanohybrid electrodes are proposed for real-time molecular detection of cancer cells, which would enable real-time tracking of H_2_O_2_ secretion in human cervical cancer cells [93]. Core-shell nanoprobes (NPs) conjugated with Mn_3_O_4_@SiO_2_ NP Mn_3_O_4_@SiO_2_(RB)-PEG-Apt, also can be used as a multifunctional nanoplatform for long-term targeted cancer imaging and therapy [94].

### 3.3. Manganese Supplementation

There are no reliable and validated biomarkers of manganese intake or status. According to European recommendations, the adequate intake (AI) for adults is 3 mg/day, including pregnant and breastfeeding women. For infants aged 7 to 11 months, the AI is 0.02–0.5 mg/day, which reflects the wide range of manganese intake that appears to be appropriate for this age group. The AI for children and adolescents is based on extrapolation from the AI for adults using isometric scaling and reference body weights of the respective age groups [95].

### 3.4. Importance of Manganese Supplementation for Cancer Treatment

Decreased Mn levels are observed in cervical cancer [96]. Mn deficiency may increase the inflammatory response, which may promote the progression of invasive adenocarcinoma of the cervix (IAC). There is a positive correlation of Mn blood levels with IL-10 and a negative correlation between IL-6 and TNF-α [97].

Mn and Se levels along with Cu/Se may be of value in patients with all histological subtypes of malignant epithelial ovarian tumors. Decreases in Mn and Se levels may be related to the adaptive defense mechanism of tumor cells directly or through AOE (antioxidant enzyme), such as Mn-SOD and GPx1, to avoid increased apoptosis. Mn levels are also important in distinguishing cancer stages. Low Mn levels may contribute to tumor growth and/or spread in serous EOC (epithelial ovarian cancer) [75]. 

Because of the observed correlation between manganese levels and the incidence and course of cervical and ovarian cancer, we suggest manganese supplementation according to European guidelines and continuing research in this direction.

## 4. Cervical Cancer

Cervical cancer is one of the most common gynaecological cancers and unfortunately, it is still one of the leading causes of death in low/middle-income countries [98]. Human papillomavirus (HPV) infection [99], early onset of sexual intercourse and multiple sexual partners, polysexuality, smoking, low socioeconomic status and past treatment for CIN II-III are crucial in the development of cervical cancer. The primary treatment is total hysterectomy with bilateral removal of the adnexa, while adjuvant treatment includes radiation therapy and chemotherapy.

### 4.1. Se and Cervical Cancer

The effects of selenium on cervical cancer and its potential mechanisms were investigated through xenograft and in vitro experiments. Xenografts of HeLa cells in female nude mice showed delayed tumour growth after intraperitoneal injection of 3 mg/kg sodium selenite (SS) for 14 days. In tumour tissue, selenium levels increased significantly after SS treatment compared to the control group. In vitro studies, SS inhibited HeLa and SiHa cell viability, blocked the cell cycle in the S phase and increased apoptosis. RNA sequencing, gene encyclopedia and Kyoto genome analysis revealed that protein O (FOXO) was an important regulatory signalling pathway for SS, which has anticancer effects. The study also showed that SS increased intracellular reactive oxygen species (ROS) and impaired mitochondrial function, which activated adenosine 5′-monophosphate (AMP)-activated protein kinase (AMPK) through phosphorylation at Thr172, resulting in activation of FOXO3a and growth arrest and DNA damage (GADD45a). Thus, SS showed anti-cancer activity, and its mechanism may be that SS is involved in inducing cell cycle arrest and enhancing cell apoptosis caused by ROS-dependent activation of the AMPK/FOXO3a/GADD45a axis [100].

Selenium nanoparticles show anticancer effects in prostate, breast, cervical, lung, colorectal and liver cancers, which are dependent on the dose administered, particle size and chemical composition. They exhibit antitumor activity due to an anti-metastatic effect by inhibiting migration and invasion processes. The effect is low expression of molecules such as cyclin D1, cyclin E and CDK2. There is also activation of apoptosis by caspase-dependent mechanisms and low expression of anti-apoptotic proteins (Bcl-2), and high expression of apoptotic proteins (Bax and Bad). Some studies link antitumor activity to the activation of cell necroptosis involving TNF and IRF1 [101]. Water-soluble and dispersed selenium nanoparticles (CPA-SeNPs) produced using CPA and Na_2_SeO_3_ in a redox reaction with ascorbic acid induce intracellular ROS increase, cell cycle arrest, cell apoptosis, membrane potential dysfunction and caspase-3 activity on HeLa cells in vitro. These results indicate that CPA-SeNPs can induce cell apoptosis HeLa cell apoptosis via an intrinsic pathway. Therefore, CPA-SeNPs can be considered a potential candidate for the treatment of cervical cancer [102]. Xia et al. [103] described selenium nanoparticles (SeNPs) decorated with hyaluronic acid (HA) (HA-SeNPs), which were used to load doxorubicin (DOX) to create cancer-targeted functionalized selenium nanoparticles HA-Se@DOX. Studies have shown that HA-Se @ DOX exhibits high activity in inhibiting HeLa cell proliferation and induces HeLa cell apoptosis through activation of the Bcl-2 signaling pathway. HA-Se@DOX exhibits more potent anti-tumor activity than free DOX and Se@DOX in vitro and in vivo. HA-Se@DOX may be a promising anticancer agent for the treatment of cervical cancer.

In contrast, CdSe quantum dots (QDs) have an inhibitory effect on Rho-associated kinase (ROCK) activity in HeLa cervical cancer cells, associated with suppression of ROCK-c-Myc signalling. Inhibition of ROCK by QD significantly reduces the stability of the c-Myc protein by reducing its phosphorylation and suppressing its activity in the transcription of target genes (e.g., HSPC111). QD treatment limits the growth of HeLa cells by reducing the ability of c-Myc to drive cell proliferation and decreasing levels of HSPC111, which is involved in regulating cell growth [104]. 

The activity of selenium compounds depends on the formation of hydrogen selenide, which is the actual trigger of cell death, so selenium-containing nucleotides may represent another option as novel compounds with anticancer therapeutic potential. Of particular note is a selenium derivative, 2′-deoxyguanosine-5′-O-selenophosphate (dGMPSe), synthesised by the oxathiaphospholane method, which causes H_2_Se release in HeLa cells but requires a different metabolic pathway involving the enzyme HINT1 (histidine triad nucleotide-binding protein1) for conversion to H_2_Se [105].

Selenium-binding protein 1 (SBP1) binds selenium, and its loss during carcinogenesis usually predicts a poor prognosis. Zhao et al. [106] showed that treatment of HeLa cervical cancer cells with selenomethionine results in increased expression of SBP1 protein, which may represent a new treatment strategy.

A mechanism for targeted delivery of selenadiazole derivative (SeD) via the SeD@MSNs-FA nanosystem has been developed to enhance the effects of radiation therapy. SeD@MSNs-FA induces in vivo antitumor effect in a HeLa xenograft nude mouse model and exhibits low toxicity. The compound is able to target cervical cancer cells and enhance their radiosensitization in vivo and in vitro. This creates the possibility of simultaneous treatment with chemotherapy and radiotherapy for cervical cancer [107]. 

Chemodynamic therapy (CDT) is a tumor microenvironment (TME)-based cancer treatment method; however, its limited amount of endogenous hydrogen peroxide (H_2_O_2_) weakens the anti-tumor effect. Zheng et al. [108] describe a multifunctional Se@SiO_2_—Mn@Au/DOX biomimetic nanozyme (named SSMA/DOX), which undergoes self-cleavage catalysis responsive to the tumor microenvironment, thus improving anti-cancer therapy.

One mouse study described RGDfC-Se @ siRNA, which silences genes in HeLa cells. It inhibits the invasion, migration and proliferation of HeLa cells, causes their apoptosis and induces disruption of mitochondrial membrane potentials. It also enhances the production of reactive oxygen species (ROS) in the HeLa cell. RGDfC-Se@siRNA thus has potential, but further research is needed [109].

### 4.2. Mn and Cervical Cancer

Manganese superoxide dismutase (MnSOD) is the main antioxidant enzyme in mitochondria, and plays an important role in protecting cells from oxidative stress, so defects in this enzyme may correlate with susceptibility to various cancers [110]. The MnSOD rs4880 polymorphism is associated with MnSOD activity. Tong et al. [111] evaluated the correlations between MnSOD genotypes and the risk of cervical carcinogenesis and the effect of serum antioxidant nutritional status (beta-carotene, lycopene, zeaxanthin/lutein, retinol, alpha-tocopherol and gamma-tocopherol). The results of the study showed that there is no link between the MnSOD rs4880 polymorphism and cervical cancer, but it was also discovered that higher levels of antioxidant micronutrients can lower the risk of CIN and cervical cancer and also modify the effect of the MnSOD polymorphism on cancer risk. Furthermore, Attatippaholkun et al. argue that polymorphisms (Val-9Ala and Ile58Thr) in the MnSOD gene are not associated with the risk of cervical cancer susceptibility and breast cancer [112].

Manganese superoxide dismutase (MnSOD or SOD2) is strongly expressed in various cancers. Rabelo–Santos et al. [113] observed that SOD2 expression is increased in cervical intraepithelial neoplasia grade 3 (CIN3) and squamous cell carcinoma (SCC) and higher in adenocarcinoma (ADC) of the cervix than in SCC. However, SOD2 expression is independent of the presence of HPV 16 and/or 18. They also conclude that the mitochondrial antioxidant system and HPV infection have an independent pathway in cervical epithelial carcinogenesis and differentiation to cervical SCC or ADC.

Manganese superoxide dismutase (Mn-SOD) is associated with the expression of the p53 oncoprotein, which affects the prognosis of patients with cervical cancer. The level of Mn-SOD is correlated with local control in tumor cells and is an important prognostic factor in radiation therapy for cervical cancer [114].

Li et al. [115] investigated that transition metal complexes based on 4-acylpyrazolone derivatives, two Mn complexes [Mn(HLa)(La)]-(CH_3_CN)1,5-H_2_O and [Mn_2_(Lb)_2_(μ -EtO)_2_(EtOH)_2_], have DNA and protein binding ability and anticancer activity. They inhibit the growth of Eca-109 esophageal cancer cells and HeLa cervical cancer cells. In contrast, Narayanan et al. [116] propose the use of phytaspase-loaded, Mn-doped ZnS quantum dots embedded in chitosan nanoparticles in cisplatin chemotherapy of HeLa cells to improve treatment efficacy.

It is claimed that an abnormal tumor microenvironment (TME), including hypoxia, acidosis and dense extracellular matrix, is associated with tumor resistance to RT. Zhang et al. [117] propose smart protein-coated nanocomposites of bismuth sulfide and manganese oxide (Bi_2_S_3_-MnO_2_) obtained by biomineralization to modulate hypoxic TME to enhance RT efficacy in cervical cancer.

Manganese dioxide nanoparticles integrated with bovine serum albumin (BSA-MnO_2_) and anchored on the surface of doxorubicin (DOX) and chlorine e6 photosensitizer (Ce6) co-loaded with hollow mesoporous silica nanospheres (BSA-MnO_2_@HMSNs-DOX-Ce6, BMHDC) can effectively inhibit human cervical cancer through synergistic therapy, as confirmed by in vitro and in vivo experiments. BSA-MnO_2_ prevents premature charge release and is an oxygen generator, through the breakdown of endogenous H_2_O_2_, which effectively deals with tumor resistance to photodynamic therapy (PDT) associated with hypoxia [118].

Studies indicate that hMnSOD-R9, or human manganese superoxide dismutase (hMnSOD) and nonamer arginine (R9), may also be beneficial in the treatment of cervical cancer. The compound induces apoptosis of HeLa cells by up-regulating cleaved caspase-3 and down-regulating the phospho-STAT3 pathway in a dose-dependent manner. It causes an increase in the expression of Bax, JNK, and TBK1 genes and a gradual decrease in STAT3 gene expression in HeLa cells [119].

## 5. Endometrial Cancer

Endometrial cancer is the most common malignant tumour of the female genital tract in developed countries [120]. Symptoms appear early so that most cancers are diagnosed at an early stage and patients’ prognosis is good [121]. Factors favouring the development of endometrial cancer include overweight and obesity, other components of the metabolic syndrome, polycystic ovary syndrome, lack of offspring, infertility, lack of ovulation, early first menstruation and late menopause, hyperprolactinaemia, use of tamoxifen, use of oestrogens in hormone replacement therapy, oestrogen-producing tumours, Lynch syndrome [122,123]. The average age of endometrial cancer is 60 years.

### 5.1. Se and Endometrial Cancer

Selenium and cisplatin conjugate (NH_3_)_2_Pt(SeO_3_) inhibits telomerase in cancer cells obtained from endometrial tumors. The presumed mode of action of this drug is the formation of free radicals that induce DNA strand breaks. Conjugate can reduce telomerase activity in cancer cells in a concentration-dependent manner, independent of sodium ascorbate [124].

Quercetin and selenium may have synergistic cytoprotective and radioprotective effects on hydrogen peroxide-induced oxidative stress in endometrial adenocarcinoma cells. The combination of quercetin and sodium selenite increases cell viability, reduces malondialdehyde (MDA) levels, decreases BAD and p53 gene expression, and shows synergistic effects in terms of gene expression [125].

Breast cancer patients treated with tamoxifen (a selective oestrogen receptor modulator) over time develop resistance to treatment and harmful oestrogen-like effects on the endometrium increasing the incidence of endometrial cancer. Methylselenic acid (MSA) can enhance the growth inhibition of 4-hydroxytamoxifene in tamoxifen-sensitive breast cancer cell lines MCF-7 and T47D. The combination of 4-hydroxytamoxifene with MSA causes even stronger growth inhibition in tamoxifen-resistant breast cancer cell lines MCF-7-LCC2 and MCF7-H2Delta16, as well as endometrial-derived HEC1A and Ishikawa cells. MSA also attenuates the induction of ER-dependent endogenous gene expression (pS2 and c-myc) by estradiol and 4-hydroxytamoxifen and ER-dependent reporter gene expression (ERE(2)e1b-luciferase) [126].

### 5.2. Mn and Endometrial Cancer

Manganese superoxide dismutase may be useful as a marker for screening endometrial cancer in patients with polycystic ovary syndrome (PCOS), so further studies should be conducted [127]. 

Highly monodisperse hollow MnO2 with a mesoporous envelope can be used as an effective nanocarrier for targeted chemotherapy of endometrial cancer. The nanoparticle inhibits the proliferation of endometrial cancer cells. Besides, the combination of H-MnO_2_—PEG/BJOE (Brucea javanica oil emulsion) shows the killing effect of BJOE on cancer cells and tumor inhibition. BJOE can also promote apoptosis in endometrial cancer by regulating protein expression [128].

The leader peptide of recombinant manganese superoxide dismutase (rMnSOD-Lp) should be used as a molecular vehicle to transportcisplatin directly into cancer cells since it delivers about four times more cisplatin to HTB-112 cells. cells treated with rMnSOD-Lp-CC show signs of nuclear and cytoplasmic fragmentation. The therapeutic effect on endometrial cancer cells is significant, while it shows minimal toxicity against normal cells [129]. 

## 6. Ovarian Cancer

**Figure 2:**
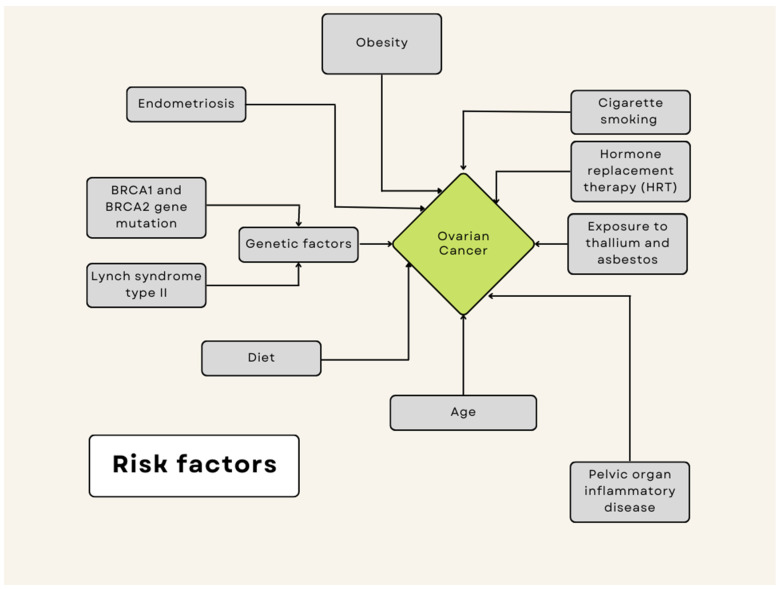
Risk factors for ovarian cancer.

Ovarian cancer is the most lethal gynecologic cancer [130], due to its very rapid onset and poor prognosis. Ovarian cancer is a neoplasm that often only manifests itself at a late stage and symptoms are often non-specific, sometimes resembling gastrointestinal symptoms [131]. It is most often detected only in stages III and IV, so early diagnosis is crucial to improve the survival of patients. The results of current therapies for ovarian cancer are not satisfactory, as they are limited by the high incidence of recurrent drug-resistant tumors, high recurrence rates and low 5-year survival rates. Risk factors for ovarian cancer include early first menstruation, late menopause [132], BRCA1 and BRCA2 gene mutations [133], obesity [134] and asbestos [135] and talc exposure. It has been suggested that chronic inflammation, oxidative stress and free radicals damage epithelial cells and what play a role in ovarian carcinogenesis [136] (Figure 2).

### 6.1. Se and Ovarian Cancer

Studies show that the incidence of ovarian cancer can correlate with various subtypes of infertility [137,138]. Endometriosis is a precursor to some ovarian cancers, especially clear cell and endometrioid cancers [139,140,141]. Antibodies to selenium-binding protein 1 (SBP1) have also been identified in ovarian cancer patients, which are characteristic of patients with premature ovarian failure (POF). This suggests that an autoimmune process may be involved in the development of serous ovarian cancers [142]. A decrease in the level of selenium binding protein 1 (SBP1) in ovarian cancer cells is observed. There is differential expression of SELENBP1 in serous borderline tumour (SBOT), micropapillary SBOT (M-SBOT) and low-grade serous carcinoma (LGSC). The observed gradual loss of SELENBP1 expression associated with increasing epithelial proliferation and papillary complexity suggests that SELENBP1 is involved in carcinogenesis. Therefore, further studies on the use of selenium in the chemoprevention and treatment of SBOT, M-SBOT and LGSC are needed [143]. 

Active selenium (Se) compounds in low doses act as prooxidants with cytotoxic effects on cancer cells, and therefore appear to be promising chemotherapeutic agents in the future. Selenium’s anticancer effects are mainly based on apoptosis [144]. Methylselenic acid can sensitize ovarian cancer cells to destruction by T cells that reduce PDL1 and VEGF levels [145]. Unfortunately, we know little about the effects of Se compounds on immune cells in the tumor microenvironment.

A major problem in chemotherapy for ovarian cancer patients is drug resistance. Selenium compounds such as selenite can prevent the induction of resistance to cisplatin or carboplatin [146]. The inclusion of selenium in treatment may also increase the effectiveness of cisplatin in inhibiting the growth of human ovarian cancer heterografts [147,148]. However, further studies are needed [149].

Selenobiotin analogs containing a selenocyanate group reduce ovarian cancer cell viability and induce apoptosis in a dose-dependent manner, as demonstrated by the cell viability assay, trypan blue dye exclusion assay, annexin V/7-AAD assay and caspase 3/7 apoptosis assay. Moreover, some showed greater efficacy than 5-fluorouracil (5-FU) and similar to cisplatin in vitro. Therefore, they have the potential for therapeutic applications in ovarian cancer patients with BR overexpression [150].

The use of nanoparticles in the treatment of ovarian cancer is proving very effective in many cases. Toubhans et al. [151] propose the use of inorganic selenium nanoparticles (SeNPs) in inhibiting the growth of ovarian cancer cells. In their work, they examined the effects of SeNPs on SKOV-3 and OVCAR-3 ovarian cancer cells. Treatment resulted in significant cytotoxicity however, the two cell types showed opposing nanomechanical responses to SeNPs, suggesting a dependence of the effects of SeNP treatment on the type of cancer cells. In contrast, Nasrolahi Shirazi et al. [152] say cyclic peptide-selenium nanoparticles could be effective transporters of drugs such as paclitaxel in the treatment of ovarian cancer.

R-Se@MEF2D-siRNAs may be an alternative treatment strategy for ovarian cancer in the clinic. R-Se@MEF2D-siRNAs have significant uptake in SKOV3 cells by clathrin-associated endocytosis. R-Se@MEF2D-siRNA can release MEF2D-siRNA faster in a microenvironment that simulates the lysosomal environment in cancer cells compared to the physiological environment. It effectively silences MEF2D gene expression in SKOV3 cells, triggers their apoptosis, has the ability to disrupt mitochondrial membrane potential (MMP) in SKOV3 cells, and causes the overproduction of reactive oxygen species (ROS). The compound also showed significant anti-tumor activity with low toxic side effects in tumor-bearing mice [153].

Selenium-chrysin (SeChry) compounds [154,155] compounds can be used to treat ovarian cancer due to their ability to produce ROS, oxidize proteins and bind DNA, leading to protein impairment, DNA damage and cell death. An encapsulated formulation of SeChry (SeChry@PURE G4-FA) is preferred, as it delivers SeChry to ovarian cancer cells (ES2, OVCAR3 and OVCAR8), reducing toxicity in non-malignant cells [156]. When using this formulation, there is also no reversal of SeChry toxicity after exposure to carboplatin.

In the paper [157], Huang et al. identify new selenium N-heterocyclic carbene (Se-NHC) compounds derived from 4,5-diarylimidazole as potential candidates for the treatment of ovarian cancer. The most active compound 2b has twice the cytotoxicity against A2780 cells than against normal ovarian epithelial IOSE80 cells. Compound 2b can also induce the production of reactive oxygen species, damage mitochondrial membrane potential, block cells in the G0/G1 phase and promote apoptosis of A2780 cells.

A synthetic selenium compound, 2-(4-methylphenyl)-1,3-selenazol-4-one, can also be used to induce apoptosis in a human ovarian cancer cell line (SKOV3) by translocating AIF, a novel proapoptotic protein [158].

A problem in the oncological treatment of gynaecological cancers is chemotherapy-induced peripheral neuropathy, the treatment of which continues to be a problem [159]. Selenium can be used to prevent and reduce chemotherapy-induced peripheral neuropathy (CIPN) in patients with platinum-sensitive recurrent ovarian cancer [160].

### 6.2. Mn and Ovarian Cancer

Manganese superoxide dismutase (MnSOD) is an important antioxidant enzyme that neutralizes the highly reactive mitochondrial superoxide radical (O^2−^) to hydrogen peroxide (H_2_O_2_), which can be further neutralised by subsequent enzymes. Excessive production of reactive oxygen species during ovulation causes damage to ovarian cells which can be a potent factor in ovarian cancer formation [161]. Therefore, it is suspected that the MnSOD polymorphism may affect the development of ovarian cancer, but further research is needed [162]. An increase in this enzyme is observed in ovarian cancer, which is a cellular response to intrinsic ROS stress [163,164]. Overexpression of MnSOD is one of the mechanisms that increase resistance to apoptosis in cancer cells. In contrast, activation of SIRT3, which regulates manganese superoxide dismutase (SOD2), is essential for anchorage-independent survival and metastasis of ovarian cancer cells [165].

Manganese-enhanced magnetic resonance imaging (MEMRI), which is currently used to assess brain activity and to track neuronal connections, may be useful for detecting ovarian cancer cells with Mn-SOD overexpression, where signal enhancement can be observed [166]. Han et al. [167] in their work described a dual-modal fluorescence/MR nanoprobe with carbon nanoparticles combined with manganese and nitrogen (Mn-N-CNSs) and conjugated to an anti-HE4 monoclonal antibody (Mn-N-CNSs @ anti-HE4) that can be used in the diagnosis of ovarian cancer cells. Chen et al. [168] present CuS-MnS_2_ nano-flowers as a multifunctional nanotheranostic agent for MRI and in combination with NIR 808 nm laser treatment a promising therapeutic agent through necroptosis for ovarian cancer.

The main obstacles to chemotherapy for ovarian cancer are chemoresistance and therapeutic selectivity. Studies show that combining drugs capable of inhibiting MnSOD with conventional chemotherapeutic agents increase the effectiveness of ovarian cancer treatment. Small interfering RNA (siRNA) on MnSOD can sensitize ovarian cancer cells to doxorubicin and paclitaxel [169]. Studies also show that ZD55-manganese superoxide dismutase (MnSOD) can sensitize human ovarian cancer cells to cisplatin-induced apoptosis [170].

Nanoparticles are playing an important role in cancer treatment today. The combination of zinc-manganese ferrite nanoparticles (PEG-MZF-NP) with CD44-shRNA, DDP (cisplatin) and magnetic fluid hyperthermia (MFH) appears to be a promising treatment for ovarian cancer [171]. Moreover, mesoporous polydopamine/MnO_2_/polydopamine nanoparticles (Pt@mPDA/MnO_2_/PDA-Z Her2) loaded with cisplatin are an alternative chemo-radiotherapy for ovarian cancer [172]. Lenis–Rojas et al. [173] evaluated the antiproliferative activity of [Mn(CO)_3_(N^N)Br] (N^N = phendione 1, bipy 3) and two newly synthesized Mn complexes [Mn(CO)_3_(acridine)(phendione)]OTf (2) and [Mn(CO) 3 (di-triazole)Br] (4) against three cancer cell lines A2780 (ovarian cancer), HCT116 (colon cancer), HCT116doxR (doxorubicin-resistant colon cancer) and in human dermal fibroblasts. In this study, the antiproliferative assay showed a dose-dependent effect higher in complexes 1 and 2 with selectivity against an ovarian cancer cell line. Exposure of A2780 cells to IC50concentrations of complexes 1 and 2 resulted in an increase in reactive oxygen species and activation of cell death mechanisms, via intrinsic apoptosis for 2 and autophagy and extrinsic apoptosis for 1. Both complexes do not damage DNA or inhibit the cell cycle but are able to enhance cell migration and neovascularization (for 2), suggesting that their use may be focused on the early stages of cancer to avoid tumor invasion and metastasis, and opening a new avenue for the use of complex 2 in regenerative medicine. Importantly, both complexes show no toxicity in both in vivo models. An interesting chemotherapeutic is the di-manganese(II) therapeutic [Mn₂(μ-oda)(phen)₄(H_2_O)₂][Mn₂(μ-oda)(phen)₄(oda) ₂]- 4H_2_O (Mn-Oda) which induces autophagy-promoted apoptosis in ovarian cancer cells (SKOV3). Mn-Oda is one of the few transition metal complexes that activate cell death only in the final stages of cytotoxicity [174].

Highlights of the effects of selenium and manganese-containing compounds on the treatment of cervical, endometrial and ovarian cancers are included in Table 2 and Table 3.

## 7. Conclusions

Gynaecological cancers are characterised by significant morbidity and mortality, but unfortunately, their aetiology is not yet thoroughly investigated. Therefore, further research into the pathogenesis of tumours is very important. Trace elements, although present in the body in negligible amounts, play a significant role in oncogenesis through their important role in inflammatory and antioxidant processes. The biological role of selenium (Se) and manganese (Mn) affects the incidence, prevalence, proliferation and mitigation of cancer. Disrupted micronutrient haemostasis can affect cell growth, division and apoptosis. The differentiation of intracellular and extracellular trace element levels in reproductive organ tumours seems to be an appropriate direction for the diagnosis and therapy of ovarian, cervical and endometrial cancer. Therefore, selenium and manganese should be supplemented according to European recommendations. The use of nanoparticles and other novel compounds containing selenium or manganese is an attractive treatment strategy for invasive cancers. On the other hand, supplementation of these elements during tumourigenesis should be considered as it can effectively counteract tumour growth.

## Figures and Tables

**Table 1:** Functions of selenium-containing enzymes.

	Function
Glutathione peroxidase	
GPx1	Antioxidative defence
GPx2	Protection against lipid peroxides resulting from lipid peroxidation
GPx3	Reduction of peroxides in plasma and other body fluids
GPx4	Reduce H_2_O_2_ and small hydroperoxides in complex lipids such as phospholipid, cholesterol and cholesterolester hydroperoxides
Iodothyronine deiodinase	
DIO1	Conversion T4 to T3
DIO2	Local production (intracellular) of T3 from T4
DIO3	Production of rT3 from T4, and T2 from T3
Thioredoxin reductases	
TrxR1	Main antioxidant “weapon” at the cellular level
TrxR2	Regulates cell proliferation
Selenoprotein	
Selenoprotein W	Antioxidant role; maintaining homeostasis of Ca^2+^ in neurons; control of expression of genes responsible for synthesis of glutathione de novo
Selenoprotein H	Antioxidant role; maintaining homeostasis of Ca^2+^ in neurons; control of expression of genes responsible for synthesis of glutathione de novo
Selenoprotein T	Antioxidant role; maintaining homeostasis of Ca^2+^ in neurons; control of expression of genes responsible for synthesis of glutathione de novo
Selenoprotein P	Selenium transporter; cooperates with the ApoER2 receptor in the transport of selenium to the brain; control of the redox potential in the cell
Selenoprotein M	Reduce isomerisation of disulphide bridges; protection of neurons against oxidative stress
Selenoprotein N	Degradation of H_2_O_2_; involved in the development of muscle tissue at an early stage of development organism; regeneration of skeletal muscle tissue
Selenoprotein K	Building protein-protein complexes; degradation of misfolded proteins on the endoplasmic reticulum (ERAD machinery); regulate the anti-inflammatory properties of selenium and its importance in the immune response
Selenoprotein S	Building protein-protein complexes; degradation of misfolded proteins on the endoplasmic reticulum (ERAD machinery); regulate the anti-inflammatory properties of selenium and its importance in the immune response
Selenoprotein V	Testes-specific expression
Selenoprotein I	Function unknown
Selenoprotein O	Function unknown

**Table 2:** Effects of selenium compounds on certain cancers.

Cancer	Selenium Form	Result	Reference
Cervical cancer	Sodium selenite (SS)	SS is involved in inducing cell cycle arrest and enhancing cell apoptosis caused by ROS-dependent activation of the AMPK/FOXO3a/GADD45a axis.	Qi et al. [100]
Cervical cancer	Water-soluble and dispersed selenium nanoparticles (CPA-SeNPs)	The CPA-SeNPs can induce cell apoptosis HeLa cell apoptosis via an intrinsic pathway.	Li et al. [102]
Cervical cancer	Selenium nanoparticles HA-Se@DOX	HA-Se@DOX exhibits more potent anti-tumor activity than free DOX and Se@DOX in vitro and in vivo.	Xia et al. [103]
Cervical cancer	CdSe quantum dots (QDs)	QD treatment limits the growth of HeLa cells by reducing the ability of c-Myc to drive cell proliferation and decreasing levels of HSPC111, which is involved in regulating cell growth.	Chen et al. [104]
Cervical cancer	Selenium derivative, 2′-deoxyguanosine-5′-O-selenophosphate (dGMPSe)	It causes the release of H_2_Se in HeLa cells, but requires a different metabolic pathway involving the enzyme HINT1 (histine nucleotide triad-binding protein1) for conversion to H_2_Se.	Krakowiak et al. [105]
Cervical cancer	Selenomethionine	The treatment of HeLa cer-vical cancer cells with selenomethionine results in increased expression of SBP1 protein.	Zhao et al. [106]
Cervical cancer	SeD@MSNs-FA nanosystem	The compound is able to target cervical cancer cells and enhance their radiosensitization in vivo and in vitro. This creates the possibility of simul-taneous treatment with chemotherapy and radiotherapy for cervical cancer.	Liu et al. [107]
Cervical cancer	Multifunctional Se@SiO_2_—Mn@Au/DOX biomimetic nanozyme (named SSMA/DOX)	It undergoes self-destruction catalysis responding to the tumour microenvironment, thereby improving anti-cancer therapy.	Zheng et al. [108]
Cervical cancer	RGDfC-Se @ siRNA	It silences genes in HeLa cells, inhibits HeLa cell invasion, migration and proliferation, induces HeLa cell apoptosis and induces mitochondrial membrane potential disruption and increases reactive oxygen species (ROS) production in HeLa cells.	Xia et al. [109]
Endometrial Cancer	Selenium and cisplatin conjugate (NH_3_)_2_Pt(SeO_3_)	It inhibits telomerase in tumour cells obtained from endometrial tumours.	Blasiak et al. [124]
Endometrial Cancer	Combination of quercetin and sodium selenite	It increases cell viability, reduces malondialdehyde (MDA) levels, decreases BAD and p53 gene expression and has synergistic effects in terms of gene expression	Cebecioglu et al. [125]
Endometrial Cancer	Methylselenic acid (MSA)	The combination of 4-hydroxytamoxifen with MSA results in even stronger growth inhibition of tamoxifen-resistant breast cancer cell lines MCF-7-LCC2 and MCF7-H2Delta16, as well as endometrial-derived HEC1A and Ishikawa cells.	Shah et al. [126]
Ovarian Cancer	Methylselenic acid	It may sensitise ovarian cancer cells to destruction by T cells, which reduce PDL1 and VEGF levels.	Nair et al. [145]
Ovarian Cancer	Selenite	It may prevent the induction of resistance to cisplatin or carboplatin.	Caffrey et al. [146]
Ovarian Cancer	Selenobiotin analogs containing a selenocyanate group	They reduce ovarian cancer cell viability and induce apoptosis in a dose-dependent manner.	Raza et al. [150]
Ovarian Cancer	Inorganic selenium nanoparticles (SeNPs)	They inhibit the growth of SKOV-3 and OVCAR-3 ovarian cancer cells.	Toubhans et al. [151]
Ovarian Cancer	Cyclic peptide-selenium nanoparticles	They can be effective transporters of drugs, such as paclitaxel, in the treatment of ovarian cancer.	Shirazi et al. [152]
Ovarian Cancer	R-Se@MEF2D-siRNA	It effectively silences MEF2D gene expression in SKOV3 cells, triggers their apoptosis, has the ability to disrupt mitochondrial membrane potential (MMP) in SKOV3 cells, and causes overproduction of reactive oxygen species (ROS).	Wang et al. [153]
Ovarian Cancer	Encapsulated formulation of SeChry (SeChry@PURE G4-FA)	It delivers SeChry to ovarian cancer cells (ES2, OVCAR3 and OVCAR8), reducing toxicity in non-cancerous cells.	Palakurthi et al. [156]
Ovarian Cancer	Selenium N-heterocyclic carbene (Se-NHC)	The most active compound 2b has twice the cytotoxicity against A2780 cells than against normal ovarian epithelial IOSE80 cells.	Huang et al. [157]
Ovarian Cancer	Synthetic selenium compound, 2-(4-methylphenyl)-1,3-selenazol-4-one	It can also be used to induce apoptosis in a human ovarian cancer cell line (SKOV3) through translocation of AIF, a novel proapoptotic protein [158].	Ahn et al. [158]

**Table 3:** Effects of manganese compounds on certain cancers.

Cancer	Manganese Form	Result	Reference
Cervical cancer	Mn complexes [Mn(HLa)(La)]-(CH_3_CN)1,5-H_2_O and [Mn_2_(Lb)_2_(μ -EtO)_2_(EtOH)_2_]	They have DNA and protein binding capacity and anti-cancer activity. They inhibit the growth of Eca-109 oesophageal cancer cells and HeLa cervical cancer cells.	Li et al. [115]
Cervical cancer	Phytaspase-loaded, Mn-doped ZnS quantum dots embedded in chitosan nanoparticles	In cisplatin chemotherapy, HeLa cells can increase treatment efficacy.	Narayanan et al. [116]
Cervical cancer	Protein-coated nanocomposites of bismuth sulfide and manga-nese oxide (Bi_2_S_3_-MnO_2_)	They can be used to modulate hypoxic TME and increase the efficacy of RT in cervical cancer.	Zhang et al. [117]
Cervical cancer	Manganese dioxide nanoparticles integrated with bovine serum albumin (BSA-MnO_2_) and anchored on the surface of doxorubicin (DOX) and chlorine e6 photo-sensitizer (Ce6) co-loaded with hollow mesoporous silica nanospheres (BSA-MnO_2_@HMSNs-DOX-Ce6, BMHDC)	BSA-MnO_2_ prevents premature charge release, and is an oxygen generator, through the breakdown of endogenous H_2_O_2_, which effectively deals with tumor resistance to photodynamic therapy (PDT) associated with hypoxia.	Fang et al. [118]
Cervical cancer	hMnSOD-R9, a human manganese superoxide dismutase (hMnSOD) and arginine nonamer (R9)	He compound induces apoptosis of HeLa cells through up-regulation of cleaved caspase-3 and down-regulation of the phospho-STAT3 pathway in a dose-dependent manner. It causes an increase in the expression of Bax, JNK, TBK1 genes and a progressive decrease in STAT3 a gene expression in HeLa cells.	Zhang et al. [119]
Endometrial Cancer	Highly monodisperse hollow MnO_2_ with a mesoporous envelope	The combination of H-MnO_2_—PEG/BJOE (Brucea javanica oil emulsion) shows the killing effect of BJOE on cancer cells and tumor inhibition.	Hu et al. [128]
Endometrial Cancer	The leader peptide of recombinant manganese superoxide dismutase (rMnSOD-Lp)	It can be used as a molecular carrier to transport cisplatin directly to cancer cells, as it delivers approximately four times more cisplatin to HTB-112 cells.	Borrelli et al. [129]
Ovarian Cancer	Small interfering RNA (siRNA) on MnSOD	It can sensitise ovarian cancer cells to doxorubicin and paclitaxel.	Yeung et al. [169]
Ovarian Cancer	ZD55-manganese superoxide dismutase (MnSOD)	It can sensitise human ovarian cancer cells to cisplatin-induced apoptosis.	Wang et al. [170]
Ovarian Cancer	The combi-nation of zinc-manganese ferrite nanoparticles (PEG-MZF-NP) with CD44-shRNA, DDP (cisplatin) and magnetic fluid hyperthermia (MFH)	Promising alternative treatment for ovarian cancer.	Guo et al. [171]
Ovarian Cancer	Mesoporous polydopamine/MnO_2_/polydopamine nanopar-ticles (Pt@mPDA/MnO_2_/PDA-Z Her2) loaded with cisplatin	Promising alternative treatment for ovarian cancer.	Wang et al. [172]
Ovarian Cancer	Di-manganese(II) therapeutic [Mn_2_(μ-oda)(phen)_4_(H_2_O)_2_][Mn_2_(μ-oda)(phen)_4_(ode)_2_]- 4H_2_O (Mn-Oda)	*It* induces autophagy-promoted apopto-sis in ovarian cancer cells (SKOV_3_).	Slator et al. [174]

## Data Availability

Not applicable.
